# Detecting stress caused by nitrogen deficit using deep learning techniques applied on plant electrophysiological data

**DOI:** 10.1038/s41598-023-36683-3

**Published:** 2023-06-14

**Authors:** Daniel González I Juclà, Elena Najdenovska, Fabien Dutoit, Laura Elena Raileanu

**Affiliations:** 1 School of Engineering and Management Vaud, HES-SO University of Applied Sciences and Arts Western Switzerland, 1401 Yverdon-les-Bains, Switzerland; 2 Universitat Politècnica de Catalunya (UPC), 08034 Barcelona, Spain

**Keywords:** Engineering, Plant signalling

## Abstract

Plant electrophysiology carries a strong potential for assessing the health of a plant. Current literature for the classification of plant electrophysiology generally comprises classical methods based on signal features that portray a simplification of the raw data and introduce a high computational cost. The Deep Learning (DL) techniques automatically learn the classification targets from the input data, overcoming the need for precalculated features. However, they are scarcely explored for identifying plant stress on electrophysiological recordings. This study applies DL techniques to the raw electrophysiological data from 16 tomato plants growing in typical production conditions to detect the presence of stress caused by a nitrogen deficiency. The proposed approach predicts the stressed state with an accuracy of around 88%, which could be increased to over 96% using a combination of the obtained prediction confidences. It outperforms the current state-of-the-art with over 8% higher accuracy and a potential for a direct application in production conditions. Moreover, the proposed approach demonstrates the ability to detect the presence of stress at its early stage. Overall, the presented findings suggest new means to automatize and improve agricultural practices with the aim of sustainability.

## Introduction

The plant’s intrinsic electrical signaling network, known as electrophysiology, is the universal mechanism that plants use to rapidly transmit the perceived stimuli from the environment through their senses to all other parts. Such a mechanism helps them react and adapt to environmental changes that manifest with variations of plant electrical potential^1^.

In recent years, by using machine learning techniques, several studies have revealed the existence of signal patterns in the monitored plant electrophysiology that could identify the plant’s health status in the presence of either biotic or abiotic stimuli^2–9^. Such studies demonstrate the potential of the monitored plant electrical response to enable an automated plant health alert system that could optimize today’s agricultural practice regarding yield and sustainability.

However, few of these studies^7–9^ explore plant electrophysiological signals acquired outside a laboratory, i.e., under typical greenhouse growing conditions, which brings a more direct impact on the everyday agricultural routine. They rely on traditional machine learning models using local signal features extracted from relatively small windows of several seconds to 30 min. One of their main aims was to study the signal characteristics and the temporal extent to which these characteristics could discriminate the state when the plant is growing in normal conditions from the stressed state caused by an applied stimulus, such as drought, nutrient deficit, or pest attack. They report accuracies of more than 80% for distinguishing these two classes.

Nevertheless, calculating features could be time-consuming and introduce an important computational cost. Moreover, as a single feature portrays a specific aspect of the signal, it is an important simplification of the raw data.

Deep Neural Networks (DNNs) have taken a dominant place in the classification field with the emergence of novel deeper architectures and access to high computing power. In addition to their high performance, another advantage of the Deep Learning (DL) methods is the ability to automatically learn classification targets from the raw input data without needing a preceding preprocessing step to generate features^5^.

Among numerous fields of application, DL techniques possess an emerging but substantial impact on improving and optimizing current agricultural practices, from production monitoring and management to robust decision support^10–14^. For instance, the use of Convolutional Neural Networks (CNNs) is the prevalent method for automated and accurate detection and classification of diverse plant diseases^15–17^. However, this diagnostic-assisting methodology is mainly based on digital images, not a measure obtained directly from the plant and, therefore, could detect the presence of the disease once the symptoms are visible.

The DL techniques have been scarcely explored for classifying the electrophysiological signal recorded from the plants. One of the reported studies in this field^6^ used two-dimensional (2D) CNNs applied to images representing the Visual Rhythm of the acquired signal^18^. Due to the limited data, the related results were not as satisfactory. More recently, another approach proposes the application of 1D CNNs to augmented data obtained from the original recordings using a Conditional Generative Adversarial Network^5^. With this approach, the accuracy for identifying salt tolerance in wheat seedlings reached approximately 93%. Nevertheless, as previously stated, both of these studies explore signals acquired in controlled laboratory conditions using a Faraday cage.

The present study aims to apply DL techniques to electrophysiology signals acquired from tomato plants growing in typical production conditions, i.e., greenhouse, to explore the ability of these advanced end-to-end classification methodologies to identify the presence of stress caused by the nitrogen deficit in the provided nutrition solution. An additional objective of the presented study is to compare the DL techniques, in terms of accuracy and inference time, against the current state-of-the-art (SOA) approaches based on more classical machine learning algorithms that require precalculated features.

## Methods

### Experimental design

The experiment for plant data collection was conducted by Agroscope, it took place at the research station in Conthey (Switzerland) starting on the 12th of July 2019. It included 16 tomato plants (*Solanum Lycopersicum*) from the commercial variety Admiro (De Ruiter) growing in a soilless manner using coconut fiber substrate. In the present study, all methods complied with the relevant guidelines and regulations. In particular, the variety used in our research is not genetically modified and does not represent a risk to the environment.

The experiment was destined to determine the effect of the deficit of the macroelement nitrogen. Normal irrigation with a complete nutrient solution was applied to each plant from the beginning of the experiment. Two-thirds of the regular nitrogen quantity was cut from the alimentation on July 18th. Visual symptoms, such as thinner stems and light-green leaves, were observed five days after the nitrogen deprivation.

The electrophysiological signal was recorded with the 8-channel device PhytlSigns (Vivent SA, Gland, Switzerland) and eight pairs of electrodes. As described in the previous studies^7–9^, the recorded signal represents the difference in the electrical potential measured between the stem, to which a ground electrode was attached, and a higher branch connected to the active electrode. It was stored at a sampling rate of 500 Hz.

### Dataset

The collected data consists of time series of univariate samples expressing the electrophysiology of each plant throughout 15 days of recordings. The first four days, corresponding to the period before the deprivation of nitrogen, represent each plant’s normal, pre-stimulus state, whereas the ten days after the application of the stimulus the stressed state.

For a more straightforward presentation, the plants from the described experiment will be further referred to with their identifiers, namely B0–B7 and C0–C7, where each letter denotes the recording device, whereas the number represents the respective channel.

**Figure 1:**
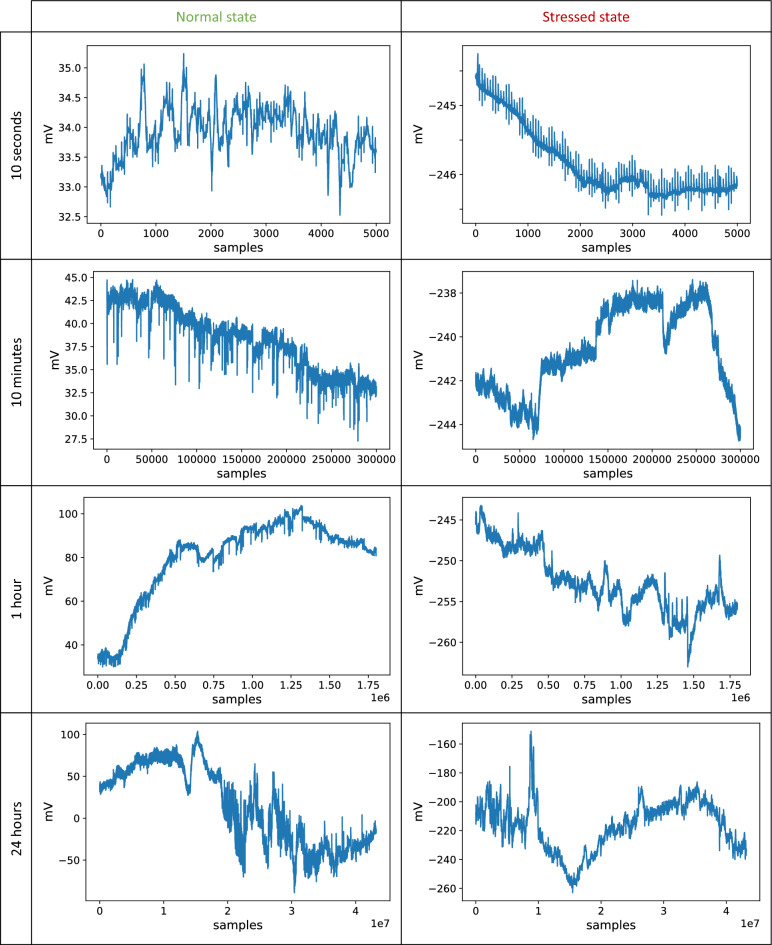
Visual representation of the electrophysiological signal recorded from a single plant for different window lengths. Each row corresponds to a distinct window length. These windows are taken either from the period when the plant was growing in normal conditions (left column) or during the stress caused by nitrogen deficiency (right column).

Initial visual data inspection showed that different segment lengths of the recorded signal portray the normal and the stressed state differently, as presented in Fig. 1. Namely, the differences between these states are more evident for windows of a few seconds than for segments of longer duration, such as several minutes or hours.

### DL architectures

A recent study^19^ presents an extensive evaluation of the performance of nine different and commonly used DL architectures for time-series classification applied on 81 specific baseline datasets. Hence, this study indicates the DL architectures most likely to achieve high accuracy for the data considered in the presented work. Among the nine, four architectures providing the highest accuracy for the largest number of the studied datasets were chosen to be tested. The following subsection briefly describes these four architectures and their implementation.

#### Multi-layer Perceptron

The Multi-layer perceptron (MLP) is a feed-forward architecture consisting of fully-connected layers with non-linear activation functions^20^. The used implementation integrates a dropout technique^21^ at each layer for regularization and rectified linear unit (ReLU)^22^ as the activation function preventing gradient saturation when the network gets deep. The network ends with a SoftMax layer to output the classification confidences for each class.

#### Fully-convolutional network

The fully-convolutional network (FCN) resides only on locally connected layers, decreasing the number of parameters to tune; therefore, such architecture requires less time to train^23^. FCN is typically used in the semantic segmentation task, where they aggregate two paths: the first, called the downsampling path, performs the extraction of data information and their interpretation, whereas the second, the upsampling one, serves for localization^23^.

As the carried-out task is classification, the used implementation encloses only the downsampling path that extracts data features at different levels of abstraction. The final classification is done with a dense layer. Moreover, the network represents a union of several blocs, where each integrates a convolutional layer followed by a batch normalization layer^24^ and a ReLU activation function.

#### Residual Network architecture

Residual Network architecture (ResNet)^25^ it’s a specific type of network formed from residual blocks, each incorporating skip connections to move over various, which enables the extension of the maximum depth of DNNs without degrading the accuracy level of the model. Such architecture allows the layers to learn the identity function considerably easier and to find an alternative path for the gradient flow, which addresses the problem of vanishing gradient in very deep structures.

The ResNet used in this work stacks three residual blocks followed by a global average pooling layer and a SoftMax layer.

#### Encoder architecture

The Encoder for time series is a CNN-based architecture that was established with the aim of building an encoder network able to learn representations that are generalizable for different types of data that were not used for the training^26^.

**Figure 2:**
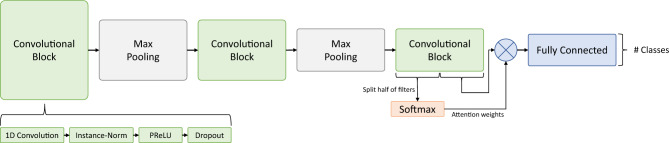
Diagram representing the Encoder architecture for time series. Adapted from Serrà et al.^26^.

In the used implementation, the CNN consists of three convolutional blocks with max-pooling layers between them. Each of these blocks is formed by a 1D convolution, followed by an instance normalization layer^27^, a parametric rectified linear unit (PReLU) activation^28^, and a dropout layer, as represented in in Fig. 2. After the last convolutional block, half of the filters are introduced as input to a time-wise SoftMax activation, that act as attention weights for the other half of the filters (Fig. 2). Finally, the result of the attention mechanism for all filters is passed through an instance normalization and a dense layer, with a SoftMax activation. Instance normalization was found to ease the training and to provide more consistent value ranges in the encoder’s output.

### Building of DL classification models

Before training the described DL architectures, the stored raw signal was filtered at 50 Hz and 100 Hz to eliminate potential power-source perturbance. The application was carried out using the official implementation provided on the respective Github repository^29^ on a machine with a GPU NVIDIA Quadro RTX 5000, CUDA 11.4, Windows 10 Enterprise, using tensorflow-gpu 2.5.0 and Python version 3.7.7.

Following the usual practice, the dataset was initially divided into a train set used for building the model and a test set for evaluating the model. To avoid eventual bias in the evaluation^8,9^, the data separation was done with respect to the plants. Namely, the samples from the plants B0, B5, and C5 formed the test set, whereas the samples of the remaining 13 plants constituted the train set. This choice of plants was randomly made.

Four days of recordings were taken from each plant to build these two sets. The first two represent the period of normal growing conditions, i.e., the normal state, whereas the remaining 48 h portray the stage when visual symptoms appeared due to the lack of nitrogen—the stressed state.

#### Choice of architecture

Within the first step of this study, preliminary modeling with the four architectures presented previously was performed to identify the most suitable algorithm, in terms of performance and accuracy, for the classification of the given plant electrophysiological data.

As previously described, all these architectures differ between them and involve a different number of parameters and layers. Hence, one should expect that the time each one would take to train is different. To make a fair comparison, the time for training for all four models was limited to 1 h and 30 min.

Signal windows with a length of 4 s were the input of each architecture, as initial exploration has shown that several seconds provide enough information to discriminate the plant states. Additionally, in preparatory analyses, similar metrics were obtained for windows of length 1 s, 8 s, and 16 s. Given that the sampling frequency is 500 Hz, each window consists of 2000 samples of the raw filtered signal.

#### Selection of hyperparameters

Several workflow designs and model parameters were explored to enable an optimized learning process during the training step.

- *Normalization type.* Through a visual exploration of the data, it was observed that the time series of each plant represents a different range of values, independently of the stressed state. Therefore, using the data without normalization could engender learning of spurious relations between the scale of a plant and the target. Another reason to normalize the data is to get better numerical stability in the training. As in the previous step, the window length used for this analysis was 4 s. The following approaches were compared:
  - *No scaling.* To have a baseline of how the model would learn without normalization, the first approach involved training with input windows without any preprocessing after filtering.
  - *Scaling per plant array*. A typical way of normalizing numeric data is scaling the input values in the range between 0 and 1, using that data’s global min and max values. The scaling employing the extrema values represents the min-max normalization. Before performing this normalization on an individual plant array, the extrema for that plant were first calculated.
  - * Scaling per window.* Another way to normalize the data is to employ the min-max normalization method on each input window individually. Such scaling would modify the structure of the time series but could also help the network not to overfit regardless of the temporal evolution of the time series. Additionally, this approach does not require knowledge about the time series data outside the given window. This normalization was done in all windows representing the data of a plant.
  - *Subtract mean per window.* The scaling performed individually on each window modifies its range and variance, which could be an important discriminative feature of the time series. To study this, another normalization approach involved only subtracting the mean value from each respective window without scaling it. In a similar manner as in the previous case, the normalization was performed for the entire data of a plant.
- *Window length.* The length of the signal sample is closely related to the extent of the discriminative information provided by the data. Consequently, the choice of window length affects the model’s performance. The selection of window lengths to explore was based on the visual data observations previously stated (Fig. 1), suggesting that the discriminative information is portrayed by windows of a few seconds. Namely, the selected candidates for tunning this parameter were 1 s, 4 s, and 16 s. Additionally, to examine the performance of longer windows with less granularity, a window length of 30 s downsampled by a factor of 3 was also considered.

### Simplification of the signal

Although the initial idea of the study was to explore the original raw signal, a visual exploration of the data resampled at a lower rate showed more evident differences between the plant’s normal and stressed states. To highlight these differences, we integrated another preprocessing step involving smoothing and resampling the signal. The employed methodology was empirically established and involved the following steps:

- *Take windows of S seconds* following a trade-off of the length to be sufficiently large to include the short signal oscillations and sufficiently small to reduce the tendency to overfitting. Such a trade-off could be established for the given data when S is set to 16 s.
- *Perform a rolling median* on the signal with a window of *N samples*. This step simplifies the signal by eliminating high-frequency variations, potentially either noise or information without a discriminating value, which could perturb the model-learning process. For the performed analyses, N was chosen to be 50, as it leads to a smoothed signal that preserves the general structure or the *envelope* of the original signal.
- *Downsample* by a *factor of F*. As the output of the previous step is a smoother signal, downsampling could be applied at this stage without losing the signal shape and structure. When F is set to 10, it enables such a result.

**Figure 3:**
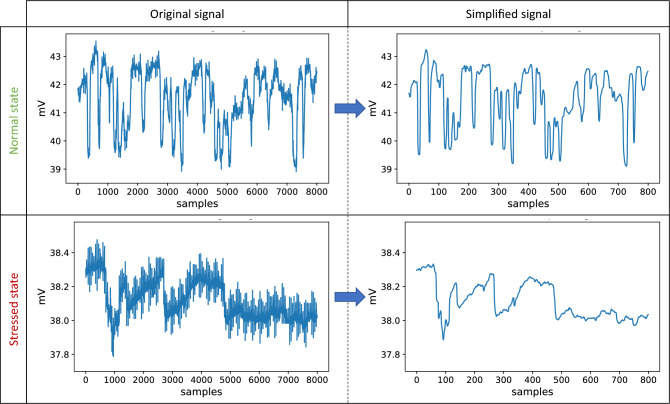
Illustration of the performed signal simplification, which includes a rolling median of 50 samples applied in 16 s signal windows, followed by downsampling of factor 10. The plots in the left column present the raw filtered data, whereas those on the right side portray the respective transformed signal. The windows from the top row correspond to the recording period without stress, whereas those from the bottom row, taken from the same plant, represent the stressed state.

Figure 3 visually represents the performed signal transformation. The steps preceding the downsampling help smooth the signal sufficiently, which is imperative for obtaining an accurate representation of its lower-frequency dynamic.

### Combination of the predictions

The particularity of the analyzed temporal series representing the electrophysiological signal of tomato plants is the absence of sudden changes from the normal state to the stressed one and vice versa. Consequently, the plant state for each sample is very likely to be the same as the neighboring samples. Hence, by combining consecutive predictions, one should expect their confidence to increase without interfering with the model providing these predictions.

The process of implementing this idea relies on two choices:

- *Method to combine the predictions*. Different functions could be used for combining the consecutive predictions. However, in this study, only the mean and median of the network’s output confidences will be considered for simplicity.
- *Length of the prediction sequence.* To make the system causal, the previous L predictions are combined to obtain the prediction for the current window. To assess the importance of the length, the chosen L values are 10 and 1000 predictions as they represent a signal of several minutes or several hours, respectively.

**Figure 4:**
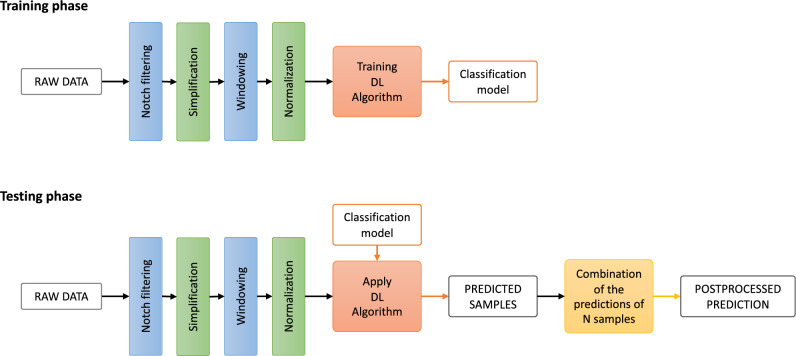
Diagram representing the entire proposed workflow for establishing the DL-based classification model. Namely, prior to training the model, the raw data undergo several preprocessing steps: notch filtering at 50 Hz and 100 Hz, simplification enclosing smoothing and undersampling, windowing to define the samples, and normalization to standardize the values range. Once the classification model was built, it was evaluated by applying it to the data from the testing set that underwent the same preprocessing procedure. An updated prediction for the entire testing set was further performed by combing the predictions of N consecutive samples.

To summarize, the entire data underwent several preprocessing steps: notch filtering, simplification, windowing, and normalization. The model was built on the training data, and its application to the testing set provided the prediction on unseen data. The prediction of N consecutive samples was further combined, which generated an updated prediction. Figure 4 shows a diagram representing the proposed workflow.

### Leave-one-out cross-validation

To get an unbiased evaluation of the model’s performance on an unseen set, a leave-one-out cross-validation (LOOCV) was performed. As the dataset encloses 16 plants, a total of 16 distinct models were built. The training set for each model included 15 different plants, and the model was evaluated on the remaining one. Prior to this analysis, the signal of each plant underwent the simplification transform described previously.

### Comparison with the state-of-the-art

The DL-based classifier was compared with the recently proposed approach by Najdenovska et al.^8^ for classifying plant electrophysiological signal based on features extracted locally from the raw data. This approach uses the XGBoost^30^ algorithm to build the classification model.

The features-extraction step requires choosing the length of the window in which the features will be calculated. The number of windows regulating the extent of the feature space is also a parameter to be selected for optimizing the classification outcome. In total, 34 features representing temporal, frequency, or time-frequency signal characteristics were calculated in each window^8^. Following preliminary studies, the highest accuracy was obtained for 15 windows with a length of 60 s, which aligns with previously reported findings^8^.

To make a reliable performance comparison, the chosen SOA approach was built and evaluated on the same train and test set as the end-to-end DL-based one. The XGBoost parameters, such as the number of trees, the max tree depth, the regularization terms lambda and alpha, and the subsampling fraction, were tuned using a custom cross-validation process where each fold was represented by the samples calculated from one plant forming the training set, as described by Najdenovska et al.^8^.

Besides the accuracy, another metric to compare these approaches is the inference time that would allow for assessing if it is feasible to predict in real-time with these models. The inference processing for Neural Networks is similar to the training as they process data in batches, which are batches of windows in the present study. These batches are processed at once for both training and inference processing. However, for training, they are all used to perform one step. Current practice often takes advantage of this to optimize the inference time when performing a prediction for more than one window, as passing one batch of N windows through the Neural Networks is faster than passing N batches of one window. Regarding the SOA approach, along with the employment of the XGBoost model, the features’ calculation is also part of the inference process.

## Results

### Building of classification models

The framework for building the classifier was set following the results for different tested scenarios.

#### Choice of DL architecture

Although 1 h and 30 min might appear as a short interval to train a DL model, the performed preliminary training was not a computing-demanding task, which allowed all four models, based on a different architecture to converge within that interval. Moreover, the encoder-based model could complete one epoch for the given time, while the other three went up to two. In fact, the encoder-based model involved a considerably larger number of parameters to learn. Table 1 lists the number of parameters used by each model, respectively. However, in terms of accuracy, the model built with the encoder architecture outperformed the other three (Table 1); therefore, this architecture will be used in the further analysis.

**Table 1:** Training and testing accuracies for the different proposed architectures.

	MLP	ResNet	FCN	Encoder
Training accuracy	55.01%	74.27%	73.94%	99.23%
Testing accuracy	52.51%	62.49%	65.12%	77.33%
# parameters	1,526,502	1,685,890	2,362,114	3,445,634

The results are obtained for windows of 4 s.

The pronounced differences between the training and testing accuracy of the built models (Table 1) indicate their tendency to overfit, which was an expected outcome since the training did not include any optimization for improving and generalizing the learning process.

#### Selection of normalization approach

The data standardization analyses demonstrated that different types of normalization affect the model performance differently. Table 2 summarizes the obtained results. More precisely, compared to the case without normalization, using any normalization approach decreases the difference between the training and the testing accuracies and improves the model performance. Despite similar results, the highest average testing accuracy is obtained with approaches that only consider the values in the given window. Additionally, the difference between the training and the testing accuracy is smaller for the min-max normalization applied to the window’s values than for the approach involving the subtraction of the mean values, which sets the choice for the normalization approach employed in the subsequent analyses. Furthermore, such an approach necessitating only the values of a given window is more easily employable in actual growing conditions.

**Table 2:** Accuracies of the models built using different normalization approaches.

	No scaling (%)	Scaling per plant array (%)	Scaling per window (%)	Subtract mean per window (%)
Training accuracy	98.25	94.66	97.87	99.10
Testing accuracy: plant B0	69.76	58.43	64.23	65.46
Testing accuracy: plant B5	96.49	90.75	96.01	97.67
Testing accuracy: plant C5	71.10	96.10	89.27	84.27
Average testing accuracy	79.12	81.76	83.17	82.47

The results are obtained for windows of 4 s.

#### Window length

Models built with windows of lengths four times smaller and four times larger than the length taken in the previous analyses (4 s) gave similar results but without achieving better classification accuracy than that obtained with the length initially tested (4 s). Such results, provided in more detail in Table 3, suggest that the window length of 4 s could be considered a local optimum for these analyses.

**Table 3:** Accuracies of the models built using different window lengths.

	1 s (%)	4 s (%)	16 s (%)	30 s (downsampled: factor 3) (%)
Training accuracy	98.83	97.87	96.36	95.75
Testing accuracy: plant B0	62.47	64.23	63.18	80.30
Testing accuracy: plant B5	95.34	96.01	98.87	99.14
Testing accuracy: plant C5	84.82	89.27	79.55	76.58
Average testing accuracy	80.88	83.17	80.56	85.34

The results are obtained using the min-max normalization per window.

However, an improvement of around 2% was observed with windows of length 30 s downsampled by a factor of 3 (Table 3). This finding suggests that data with less granularity could portray more enhanced discriminative information that would furthermore improve the model performance, which is in line with previous visual observations leading to the analysis using the simplified signal.

#### Simplification of the signal

The use of the simplified signal considerably reduces the models’ tendency to overfit. Namely, as shown in Table 4, the training accuracy is decreased and more similar to the testing accuracy of the model. Moreover, the testing accuracy is higher than in the previously analyzed cases, and the difference between the individual testing accuracies is lower.

**Table 4:** Accuracies of the model built using the simplified signal.

	Simplified signal (%)
Training accuracy	88.94
Testing accuracy: plant B0	83.40
Testing accuracy: plant B5	92.42
Testing accuracy: plant C5	87.08
Average testing accuracy	87.57

The results are obtained using the min-max normalization per window.

#### Combining the predictions

The proposed approach for combining the prediction confidence improved testing accuracy by around 10% when using the predictions of the 10 previous samples. The combination with 1000 samples provided even higher accuracies, around 99%. The results are more detailed in Table 5.

The combination employing the mean function for both sequence lengths resulted in similar accuracies to the combination with the median. Hence, as the prediction distribution is not expected to be skewed, the combination with the mean will be used in the further analyses.

**Table 5:** Accuracies obtained for different tested approaches for combining the sample-prediction confidence.

	Without combining (%)	10 samples		1000 samples	
		Median (%)	Mean (%)	Median (%)	Mean (%)
Training accuracy	88.94	95.27	96.43	99.24	99.24
Testing accuracy: plant B0	83.40	92.60	92.88	99.30	99.30
Testing accuracy: plant B5	92.42	98.65	99.03	99.77	99.77
Testing accuracy: plant C5	87.08	97.05	97.84	97.92	97.92
Average testing accuracy	87.57	96.10	96.59	99.00	99.00

#### Leave-one-out cross-validation

The LOOCV analysis demonstrated stable training accuracy across all 16 models, averaging 83.21% with a standard deviation lower than 1%. Nevertheless, high variability with a standard deviation of around 12% was observed across the testing accuracies, engendering a difference of around 6% between the mean training and testing prediction. Table 6 details these results. Although individual testing accuracies are relatively high in most cases, the prediction for plants B3, B4, and C6 is below 60%.

The combination of 1000 predictions was also performed for each individual case, which improved the average testing accuracy by around 10%. However, the outcome for B3, B4, and C6 did not present a notable improvement.

**Table 6:** The results obtained from the LOOCV analysis.

Validation Plant (Plant left out of training)	Training accuracy (%)	Testing accuracy (without combination) (%)	Testing accuracy after combining 1000 predictions (%)
B0	83.49	80.97	99.91
B1	83.56	81.92	99.97
B2	83.45	69.47	70.22
B3	84.31	56.26	64.43
B4	84.24	56.42	63.36
B5	84.24	90.21	99.91
B6	82.77	84.19	99.92
B7	82.59	84.57	99.98
C0	82.03	90.68	99.97
C1	83.92	68.79	74.99
C2	83.30	76.39	89.61
C3	82.91	87.97	99.94
C4	82.45	78.70	99.72
C5	82.17	87.19	97.20
C6	83.31	53.30	49.98
C7	82.71	85.07	92.91
Average	83.21	77.00	87.62
Standard deviation	0.71	12.10	15.97

### Application of the model to the full-length recordings

**Figure 5:**
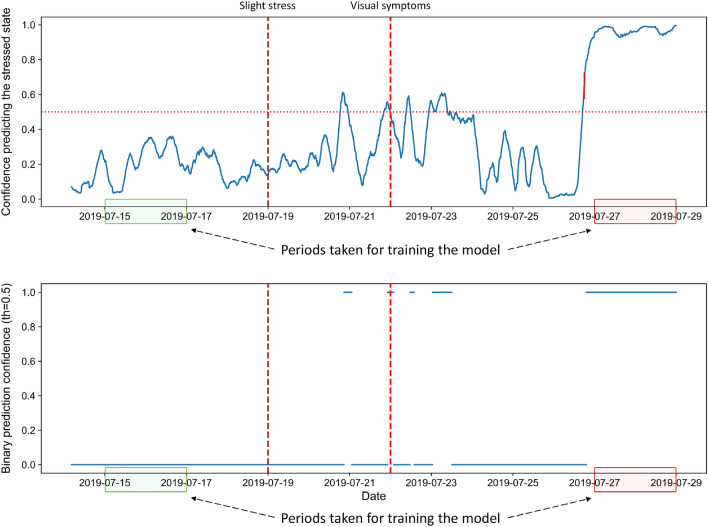
Prediction confidences of the testing plant B5. The top plot shows the raw confidences for the prediction of each sample, whereas the bottom plot gives the same confidences thresholded by 0.5. The vertical dotted lines indicate the beginning of the respective stress stages, whereas the green and red areas indicate the time intervals from the normal and stressed state, respectively, taken from training plants’ recordings to train the model.

To analyze the behavior of the built end-to-end DL-based classifier for different stress stages, it was applied to each individual full-length electrophysiological signal recorded from the test plants. Figure 5 illustrates the obtained results for one plant, showing confidence in predicting each sample’s stress state.

One could observe that the model performs well for the time intervals used for training. In contrast, the model often fails to identify the stressed state for the period during early stress and the beginning of the appearance of visual symptoms. Such observations are more evident on the plot showing the thresholded confidence in Fig. 5. For certain samples representing the stress at its early stage, the model is able to detect the presence of stress with a confidence higher than 0.5, but, in general, the prediction confidence is as low as for the samples from the normal state.

These findings suggest that plants react differently at different stages of stress. Hence, the model should be trained on data representing more extended time intervals to learn a broader range of variabilities characterizing stress.

An additional model was therefore built applying the same workflow established in this study that was used for the previous model. The new model was trained using 216 h from each plant, where half represented the normal state and the other half the stressed state.

**Figure 6:**
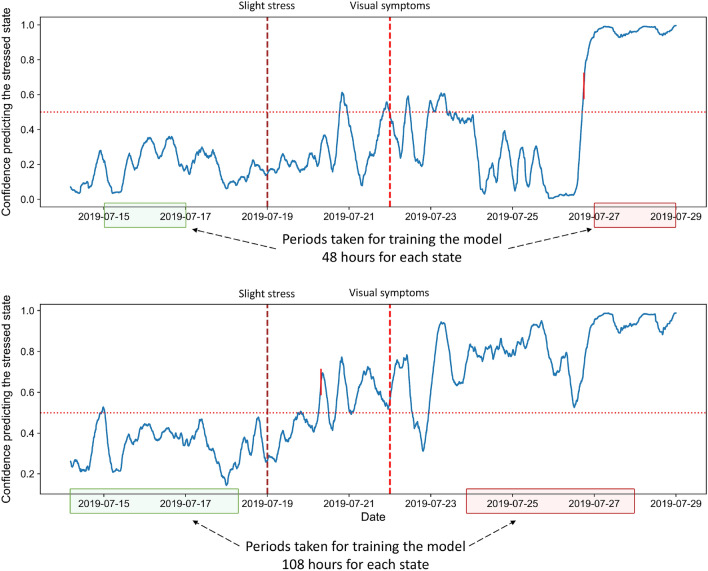
Comparison between the prediction confidences from the models built for different signal lengths for the same testing plant. The top row shows the plot of the prediction confidences obtained when applying the model trained with 48-h recordings for each state on the testing plant B5. The bottom row gives the prediction confidence for the same plant but obtained from the model trained with 108-h recordings from each state. The vertical dotted lines indicate the beginning of the respective stress stages, whereas the green and red areas indicate the time intervals from the normal and stressed state, respectively, taken from training plants’ recordings to train the model.

**Figure 7:**
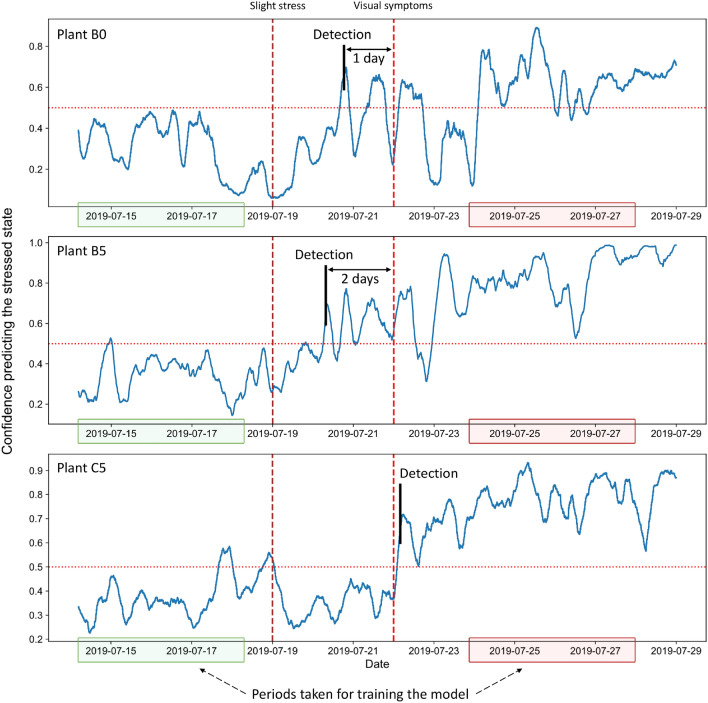
Prediction confidences of the three testing plants (B0, B5, and C5) obtained with the model trained on extended recordings length. The vertical dotted lines indicate the beginning of the respective stress stages, whereas the green and red areas indicate the time intervals from the normal and stressed state, respectively, taken from training plants’ recordings to train the model. The vertical black marker labeled ”Detection” indicates the temporal point at which the model predicts the presence of stress with high enough confidence.

As expected, the model built on extended signal recordings could more accurately predict the presence of stress throughout its different stages. Namely, the confidence for predicting the stressed state is higher than 0.5 for the main part of the recordings acquired after applying the stressor. The confidence for predicting stress during the normal state is also increased but generally remains below 0.5. Figure 6 visually represents these observations for one of the testing plants. Moreover, Fig. 7 gives the obtained prediction confidence for all three plants from the test set.

Generally, with the built model on an extended length of recordings, it is possible to detect the presence of stress before it is very pronounced or visually apparent. With the resulting prediction confidence, the beginning of the stressed state could be considered the first peak of its temporal evolution that is higher than a given threshold above 0.5. For a threshold value set to 0.65, the presence of stress for plants B0 and B5 is detected 1 to 2 days before these plants showed visual symptoms related to the nitrogen deficiency (Fig. 7). In the case of plant C5, the model detected the stressed state on the day when the deficiency started being visually noticeable (Fig. 7).

### Comparison with the state-of-the-art

The model built with the XGBoost algorithm applied on signal features extracted from the electrophysiological recordings, as proposed by Najdenovska et al.^8^, predicts the stress on the testing plants with an accuracy of 79.27%, which is similar to previously reported findings by the same authors for classification of plant electrophysiology^8^. However, comparing it to the results from this study summarized in Table 5, the proposed DL-based approaches considerably increase the prediction accuracy. Solely the DL model built on the simplified signal performs with around 8% higher testing accuracy, whereas after combining its predictions, the differences reach over 17%.

The inference time for both approaches was computed for a single window and within a batch of 64 windows, as reported in Table 7. One could observe that, compared to a batch of a single window, processing in a batch of 64 windows is three times faster in CPU and ten times in GPU.

In GPU using a batch of 64 windows, the prediction with the DL-based model takes 0.00057 s. The prediction with the XGBoost for 15 windows of 60 s, which are the parameters used for this experiment, takes 0.0156 s if the sample is predicted alone. If instead, it is predicted in a batch of 64 samples, it takes only 0.00028 s per sample. Hence, the time for performing the prediction only is similar to the DL-based approach. However, as the calculation of the features of 15 windows takes 3.73 s, the total inference time of the SOA method is considerably increased as the preprocessing step dominates it.

**Table 7:** Comparison between the inference time of the SOA approach requiring features-extraction step and the proposed end-to-end DL-based approach.

	DL with CPU	DL with GPU	XGBoost	Feature extraction + XGBoost
Batch of 1 window	0.0210 s	0.00540 s	0.01560 s	3.75 s
Batch of 64 windows	0.0074 s	0.00057 s	0.00028 s	3.73 s

## Discussion

This work presents an end-to-end DL-based approach, using the encoder architecture for time series, that highly accurately classifies plant electrophysiological signal acquired within the scope of studying the effect of nitrogen deficiency on tomato plants growing in typical greenhouse conditions. The developed workflow directly explores the raw acquired signal, eliminating the need for a preprocessing step calculating signal features, which is required by the current SOA techniques based on classical machine learning algorithms.

Applied on a simplified signal representing 10 times less granularity than the original signal stored at 500 Hz, the proposed DL methodology predicts the presence of stress due to lack of nitrogen with an accuracy of around 88%. Without prior knowledge regarding the frequency band that potentially represents the information with a more discriminative power, we initially aimed to explore the raw signal at the original sampling rate. However, the model built on the simplified signal leads to an improved prediction regarding the initial models built on the original raw data. Additionally, using the downsampled smoothed signal considerably reduces the models’ tendency to overfit, which is represented by the large difference between the training and the testing accuracy in the previous cases. These findings suggest that the signal variations at high frequencies, potentially representing noise, portray high variability between plants, which perturbs the learning of the model. Along with the proposed methodology for simplifying the data, the granularity of the simplified signal was empirically established, but future studies could investigate further the frequencies to identify those that portray the patterns discerning the different states.

The inter-plant variabilities are predominantly evident from the LOOCV analysis, showing large differences across the individual plant testing accuracies. Additionally, in less than 20% of the cases, the respective model, although with high training accuracy, performed a poor prediction. These differences could be due to the individual plant reaction to the applied stressor, closely related to its latent biological and health predisposition. Namely, while most plants would endure critical stress caused by the nitrogen deficiency after a particular time, some could be more resilient or would need a longer time interval to present the same reaction. Nevertheless, as the training accuracies remain stable across all 16 models, we can assume that the observed variabilities are immersed through the learning phase. Hence, a larger number of plants should be further considered allowing the model to learn and enclose a broader spectrum of these variabilities.

The normalization of the data compensates for the range-based inter-plant variabilities. Moreover, the chosen standardization methodology is dependent only on a given window’s values, without needing to know the values range and evolution of the entire recorded time series. Hence, it enables a more straightforward application of the developed workflow for predicting the plant status in actual growing conditions. The assessed inference time of order of ms fortifies the possibility for such an application.

The combination of the prediction confidence for the last subsequent samples leads to a substantial increase in the model’s accuracy. The prediction is improved by around 10% for a sequence of only 10 samples, whereas for 1000 samples, it reaches 99%. Nevertheless, the system would need more time for a large sequence to realize the change from one state to another. For instance, given that the selected window length is 16 s, the prediction delay for a combination of 1000 samples is 4.44 h, whereas for 10 samples is only 2.66 min. However, as the plant’s reaction to the nitrogen deficiency is a relatively slow process, detecting the presence of stress after four and a half hours could still be acceptable for the growers if the accuracy of that prediction is crucial.

The fusion decision based on previous outputs to improve the prediction accuracy is a concept introduced previously. The literature proposes numerous techniques to this end, mainly used for combining the output of different single learners in an ensemble model incorporating the advantages of the individual learners, therefore achieving better performance^31^. Moreover, confidence-based fusion has been employed within classification frameworks for different physiological signals^32,33^. However, different from these studies, our method uses the output from the same algorithm but for subsequent samples relying on the assumption that the likelihood of quick variation between the states is very low. Consequently, this approach is more straightforward than the usual combination of outputs from different learners.

The presented findings also show the strong potential for detecting the presence of stress in its early stage before the plant manifests evidently the lack of nitrogen. Such detection was notably more achievable for models trained on extended recordings intervals that allow the model to learn different plant reactions concerning different stress intensities. Early stress detection would potentially mitigate the damage and require fewer resources to increase and preserve the crop’s health. The threshold of 0.65 detecting the first confidence peak is selected empirically for the plants forming the used test set in this study. However, it could be further studied for an extended set of plants to establish a value that better generalizes inter-plant variabilities regarding the reaction to the applied stressor.

The proposed methodology outperforms the current SOA approach in the field based on more classical machine learning techniques^8^ by achieving higher accuracy of around 8% without applying the combination of the predictions, and 177 to 6544 times faster inference processing, depending on the configuration of the accelerator and the batch size. This important difference is due to the DL-based approach’s absence of the features-extracting process. However, it is worth noting that the time required to calculate the features depends on how computationally demanding they are. Furthermore, reducing the feature set by selecting the most discriminative ones could also decrease the inference time for this preprocessing step. Nevertheless, the ranking provided by the XGBoost gain measure indicates that among the three most discriminative features, one is related to wavelet decomposition, which takes considerably longer to compute than all other features. Hence, for the studied data, reducing the feature space to the set enclosing the most discriminative ones would not sustainably change the inference time for their extraction. However, the discriminatory information is closely related to the applied stimulus, i.e., the reaction it provokes in plants. Therefore, the inference time for the SOA approach could be much lower when classifying electrophysiological data acquired from plants that undergo different stress. On the other hand, as DL algorithms can learn complex patterns and relationships that may not be immediately apparent to traditional techniques, they could be more suitable for classifying plant electrophysiological data enclosing different stresses caused by multiple distinct stimuli and, therefore, portrayed by different signal patterns that, for traditional machine learning techniques, would require the extraction of numerous sets of features to represent them.

The calculation of the features predominates the inference time of the SOA approach. However, as the features represent the information on which the model is built, their extraction is essential for performing the prediction. Moreover, the SOA approach employs a normalization methodology requiring the knowledge of the full-length signal, and not only the given window, to deduce the needed extreme values, which deployment for plant state prediction in everyday agricultural practice is more challenging. An approximation of the plant range could potentially be established, but such an approach would introduce a bias.

The proposed approach should further be validated for an extended set of plants, which would also enlarge the generalizability of the built model. Additionally, given that the studied data portrays highly discriminative information, the developed framework could be extended toward a multi-class classification predicting different stages of stress caused by a lack of nitrogen. Moreover, to assess the universality of the presented findings, the framework could be further applied to different types of stressors or different crops.

Overall, the present work provides a new tool for fast and accurate identification of nitrogen deficit in commercial tomato plants based on the raw recorded electrophysiological signal, outperforming the current literature and allowing stress detection at early stages. Moreover, the proposed methodology possesses strong potential for a direct application in production conditions, enabling new paths toward an automated agricultural practice.

## Data Availability

The data presented in this study are not publicly available due to the commercial interests of Vivent SA. However, at the sole discretion of Vivent, access may be granted to interested parties by contacting Nigel Wallbridge of Vivent SA at research@vivent.ch or nigel.wallbridge@vivent.ch.
